# Correspondence

**Published:** 1860-07

**Authors:** 


### Correspondence.

MESSRS. EDITORS:—New York city—it may not be generally known—is a place of some note, and is situated on the Island of Manhattan, near the town of Harlem.

It contains among its population *several* dentists, some of whom entertain the very singular notion that dentistry, as practiced there, is ahead of all creation, and that it is the Mecca where all that seek advancement or improvement, professionally, must come to worship.

Among the comparatively recent discoveries claimed as having been made there or thereabouts, may be enumerated *Sponge Gold*, *Vulcanite*, *Coralite*, and *Oxide of Zinc Fillings* under various startling and attractive names, such as Odonplasma, Osteoplastic, Os-artificial, etc., etc.

The first is nearly worked out, so far as I can ascertain; the second is working up; the third has no defender since its discoverer left for new fields of culture, "fancy free;" the fourth is in a transitory condition, but earnestly advocated by its respective forefathers (for it seems to have had *four* or more), and very recently recommended by the publication in a dental journal, of an attractive article containing an exceedingly picturesque history—verging on the sublime—of its rise and progress; a history that reads like a romance, and that is calculated to fire the blood and intensify the imagination, reminding one of the march of the simoon in its flight —self-progressive and surmounting all obstacles that present themselves in its course; and in which is mapped out its track and happy approach to our hospitable shores and the protecting and fostering care of the great American eagle.

As a sample brick, here is the first and a portion of the second paragraph of the article referred to:

"*Os-artificial Fillings.*—The *debut* of novelties, like the *debut* of players, is hailed with great gusto by the community or profession that is supposed to be benefited by them. The halo that surrounds them during their state of novelty gradually disappears, when they assume a reality. Man, as an imaginative being, loves to look upon all things as they appear, not as they really are—a bare fact, a thing real, is but a skeleton; the vivid imagination throws in it and around it the viscera, blood-vessels, muscles, and vitality, and dresses it in a garb of beauty, and falls at its feet and unconsciously worships a thing of its own creation."

So much for *man* and his idiosyncrasies. Now for the *history*.

"Now, just ten years past the middle of the nineteenth century, a new thing has dawned upon the dental profession. It rose, as a bright star, far in the north of Germany, swept rapidly southward over Prussia, southern Germany, and France, and, like the German emigrant, scouring England's shores, struck boldly across the broad Atlantic to America, the father of dentistry, where it received a hearty welcome, and has already become vastly improved, and is rapidly passing from its state of novelty to a thing real. It has won its way in nearly every dentist's office, from the Atlantic to the Pacific; and I venture to say no one who has tried the *material, properly prepared*, with an unbiased mind, would be willing to do without it," etc.

Please note how graceful the descent from the beautiful to the useful. Now, may I not ask, is not science eloquent, when a "*material properly prepared*" for temporary fillings should supply such an inspiration as is here evidenced?

In addition to the Quarterly Journal, which has been published for some time in New York, we have now "The Vulcanite," a new Quarterly, devoted to "Hard rubber" and dental mechanics generally, a worthy object, and therefore deserving success. But this is not all that distinguishes New York, for on a recent visit to this "proud Empire City," as a recent dental orator was pleased to term it, I had the pleasure of meeting an old friend,—a pioneer in the profession, both here and in Europe, a *dental millionaire* (what other city can boast of such?) whose annual income, I learned incidentally, was about eighty thousand dollars—not from his profession, be it understood, for he does not now practice, his son having succeeded him. I might violate the proprieties by giving the name, but will attempt a pen and ink sketch.

Picture to yourself a fatherly looking gentleman of, say sixty-five years; over an average hight, say five feet ten inches, well built, well kept, and well conditioned, but not fleshy, and as straight as an arrow; gray hair and large gray beard, patriarchal in extent, well marked features, forming an intellectual face; as earnest a manner, as elastic a step, and with as much apparent vitality and vigor as a youth of twenty, add to which, a peculiarly prompt and hearty recognition of friend or acquaintance, and you have an outline of the appearance and manner of the man before you. Do you recognize the picture?

*Hard Rubber.*—I had some additional evidence, while there, of the availability of this substance as a base for teeth, particularly as regards its strength, and of its increasing use by the prefession. I have suggested before in this correspondence, that the only apparent serious objection to it as a base was its dingy color, or imperfect representation of the natural gum, but this I was informed, it was expected would be remedied by experiments then in progress, and which, it is to be hoped, may be successful.

I very much regret to learn that Prof. Harris, who has been quite unwell for some months, is still unable to give any attention to his professional duties; and I am quite sure that all will join me in the hope that he may speedily recover, and be enabled to continue in the good work in which he has so earnestly and efficiently labored, that of professional progress.

*The Japanese* are now in this city, and of course, are all the rage. On Sunday, June 10th, the medical men attached to the Embassy were taken to witness a surgical operation for lithotomy, performed by Prof. Gross, assisted by a large number of sanguinarians. They are said to have been quite delighted with the bloody performance, and to have examined with great care and admiration the instruments employed, etc.

It will be remembered that while in Washington, the physicians there and these Japanese doctors had quite a *pow-wow*, and that the latter were inducted into the use of numerous *potent* remedies, including the high art of surgery.

Now, in view of these facts, do not your sympathies run out toward the poor inhabitants of Japan, when you reflect that on the return of these, their doctors, they shall have inaugurated the *heroic* practice learned of their more scientific and civilized brethren of North America? What a change! From laxatives to the lancet; from poultices to poisons; from simple remedies to surgical operations. The picture is too sad to contemplate without pain, and I forbear. Yours,

PHILADELPHIA, June 13, 1860. O. U. C.

